# Development of Chitosan Polysaccharide-Based Magnetic Gel for Direct Red 83:1 Removal from Water

**DOI:** 10.3390/gels10080496

**Published:** 2024-07-26

**Authors:** Ainoa Murcia-Salvador, María Isabel Rodríguez-López, José Antonio Pellicer, Teresa Gómez-Morte, David Auñón-Calles, María Josefa Yáñez-Gascón, José Pedro Cerón-Carrasco, Ángel Gil-Izquierdo, Estrella Núñez-Delicado, José Antonio Gabaldón

**Affiliations:** 1 Molecular Recognition and Encapsulation Research Group (REM), Health Sciences Department, Universidad Católica de Murcia (UCAM), Campus de los Jerónimos 135, E-30107 Guadalupe, Spain; amurcia6@alu.ucam.edu (A.M.-S.); mirodriguez@ucam.edu (M.I.R.-L.); japellicer@ucam.edu (J.A.P.); tgomez@ucam.edu (T.G.-M.); daunon@ucam.edu (D.A.-C.); mjgascon@ucam.edu (M.J.Y.-G.); enunez@ucam.edu (E.N.-D.); 2 Centro Universitario de la Defensa, Universidad Politécnica de Cartagena, C/Coronel López Peña s/n, Base Aérea de San Javier, E-30720 Santiago de la Ribera, Spain; jose.ceron@cud.upct.es; 3 Research Group on Quality, Safety and Bioactivity of Plant Foods, Department of Food Science and Technology, CEBAS-CSIC, University Campus of Espinardo-Edif. 25, E-30100 Espinardo, Spain; angelgil@cebas.csic.es

**Keywords:** chitosan, adsorption, isotherm, kinetic, direct red, magnetic

## Abstract

Water pollution caused by dyes is a significant environmental issue, necessitating the development of effective, cost-efficient decolorization methods suitable for industrial use. In this study, a Chitosan-Fe polymeric gel was synthesized, characterized, and tested for removing the azo dye Direct Red 83:1 from water. The polymeric magnetic chitosan was analyzed using various techniques: Field Emission Scanning Electron Microscopy (FE-SEM) revealed a porous structure, Differential Scanning Calorimetry (DSC) and Thermal Gravimetric Analysis (TGA) demonstrated the thermal stability, Infrared Spectrophotometry (IR) indicated the successful coordination of iron at the C3 position, and X-ray Powder Diffraction (XRD) confirmed the crystalline nature of the polymeric structure. Optimal conditions for kinetic and isotherm models were found at 1 g and pH 7.0. Adsorption behavior of Direct Red 83:1 onto magnetic chitosan gel beads was studied through kinetic tests and isotherm curves. The maximum adsorption capacity was 17.46 mg/g (qmax). The adsorption process followed pseudo-second-order kinetics (R^2^ = 0.999) and fit the Temkin isotherm (R^2^ = 0.946), suggesting heterogeneous surface adsorption. The newly synthesized Chitosan-Fe polymeric gel demonstrated good adsorption properties and facilitated easy separation of purified water.

## 1. Introduction

The world has witnessed a rapid increase in the global population, industrialization, agricultural activities, and unplanned urbanization, along with the excessive use of chemicals. Unfortunately, these factors have collectively contributed to a concerning issue of environmental pollution. One significant consequence is the release of colored wastewaters containing dyes from various industries such as the textiles, paper, leather, printing, plastic, and food processing [1].

The presence of dyes in textile wastewater poses a serious environmental challenge due to their inherent characteristics like resistance, high visibility, non-biodegradability, and considerable toxicity. Moreover, there is a potential risk of these dyes transforming into carcinogenic, teratogenic, and mutagenic substances, amplifying the concern even further [2]. For centuries, dyes have found extensive use in applications such as paint and textiles due to their ease of application, vibrant colors, and water-fastness [3]. However, their usage comes with detrimental consequences, including adverse health effects on humans, leading to issues like cancer, jaundice, tumors, heart defects, skin irritation, allergies, and mutations [4]. Moreover, the release of these dyes into environmental water bodies results in significant challenges for both aquatic and terrestrial ecosystems, such as interfering with photosynthetic processes in aquatic plants, decreasing oxygen levels in water, and, in extreme situations, leading to the suffocation of aquatic fauna and flora [3]. Addressing these concerns has become a vital priority for protecting the environment and human health.

Commercial dyes are typically classified according to various factors, such as their color, chemical structure, chemical nature, and application method. Additionally, based on the charge they carry upon dissolution in an aqueous medium, dyes are categorized as cationic, anionic, or non-ionic. Azo dyes pose a significant threat due to the presence of amine groups in the effluent [5]. These dyes are known to be toxic and highly hazardous to both environmental life and human health. Consequently, dye industries have faced mounting pressure to effectively eliminate or minimize the discharge of dyes into water streams before wastewater is released. Such measures are essential to safeguard the environment and protect human well-being from the potential harmful effects of these dyes. As a result, it becomes imperative to carefully choose an appropriate treatment method to effectively remove dyes from wastewater and enhance the quality of treated water released into the environment. Consequently, numerous techniques have been explored and applied to tackle the challenge of dye wastewater treatment [6].

The removal of dyes from water is crucial for safeguarding human health and ecosystems. Developing efficient decolorization techniques has practical implications for industrial processes and wastewater treatment. Among the numerous methods utilized, prevalent techniques encompass adsorption on activated carbons, coagulation and flocculation, reverse osmosis, chemical oxidation, activated sludge, bacterial action, ozonation, membrane filtration, ion exchange, and electrochemical methods, all designed for the elimination of dyes from wastewater. [7]. However, these methods have shown certain limitations, such as high operational costs, limited effectiveness, generation of excess sludge, strict environmental requirements, or the possibility of producing more toxic byproducts [7].

Of all these techniques, adsorption stands out as a highly effective approach for dye removal due to its straightforward design, high efficiency, affordability, production of non-toxic byproducts, rapid adsorption rate, and versatile applicability. Recently, there has been a growing focus on materials based on natural polymers, which offer the advantage of removing pollutants from contaminated water at a reduced cost [8]. By exploring and utilizing these natural polymer-based materials, progress can be made in addressing the challenges associated with dye wastewater treatment in a more sustainable and efficient manner [8,9,10,11,12].

In this way, chitosan polysaccharide-based gels have received considerable attention over the last twenty years as efficient adsorbents of heavy metal ions and dyes from wastewater [9], due to the presence of highly reactive amino and hydroxyl groups in its chemical structure, making it able to interact with atoms or molecules and increasing the adsorption capacity of chitosan over other biopolymers. Notably, it exhibits a high adsorption capacity, macromolecular structure, abundance, simpler functionalization and polymerization process, and cost-effectiveness [10,11]. Chitosan, also known as poly-(1-4)-2-amino-2-deoxy-β-D-glucose, ranks as the second most abundant polysaccharide globally. It is synthesized through the deacetylation of chitin, a substance abundantly found in the shells of shellfish (crabs, lobsters, prawns, crayfish), fungi, insects, and other crustaceans. This feature makes chitosan a sustainable and eco-friendly choice [13].

The chitosan biopolymer possesses several noteworthy characteristics: it is heterogeneous, linear, cationic, and boasts a high molecular weight. Its hydrophobic nature contributes to its exceptional adsorption properties. Additionally, chitosan is non-toxic, biodegradable, hydrophilic, and biocompatible, making it even more desirable for various applications, including wastewater treatment [14]. These properties collectively position chitosan as a versatile and effective material for addressing dye pollution challenges while being environmentally friendly. It has been used in recent years to remove anionic dyes like the acidic black-172 [15], or blue-25 [16], the reactive brilliant red [17], or red–195 [18], and cationic ones such as basic bright yellow [19], methylene blue [20], or malachite green [21] from water solutions.

Despite chitosan’s advantages, it has some drawbacks, such as lower absorption sites, porosity and surface area, as well as difficult separation after the absorption process, which restrict its widespread use as adsorbent [22]. To overcome these disadvantages, modified chitosan-based adsorbents with physical or chemical cross-linking, grafting or impregnation with different reagents [14], atoms coordination [22], addition of fillers such as graphene oxide [23], or iron magnetic nanoparticles in the chemical structure of chitosan have been developed, increasing their dye absorption capacity [24].

Thus, magnetic chitosan hydrogels refer to the incorporation of Fe magnetic nanoparticles into the chitosan polysaccharide gel structure. These can be synthesized by different approaches (hydrothermal, laser pyrolysis, micelles, thermal decomposition/reduction, and co-precipitation), with the latter being the cheapest and simplest method [25]. This gives rise to what some authors have recently called ferrogels [26], with applications in medicine [25], analytical diagnostics, and environmental remediation [25].

For instance, Jamali and Akbari [22] fabricated magnetic chitosan hydrogel beads modified by interfacial polymerization, demonstrating high efficiency in removing both cationic and anionic dyes from aqueous solutions and easy separation using an external magnetic field. Similarly, Singh et al. [23] developed chitosan-graphene oxide hydrogels embedded with magnetic iron oxide nanoparticles, which exhibited improved adsorption capacities due to the synergistic effects of chitosan and graphene oxide, providing a large surface area and multiple functional groups for dye interaction. Additionally, Hingrajiya and Patel [24] synthesized an Fe_3_O_4_ modified chitosan-based co-polymeric magnetic composite hydrogel for the removal of methylene blue, showcasing high adsorption capacity and rapid separation facilitated by its magnetic properties. In these studies, the composites were habitually prepared by using an ex-situ approach, in which previously synthesized magnetic particles were functionalized with chitosan and other cross-linking agents.

Considering the advantages and good performance of chitosan as a versatile adsorbent for dye removal, continued effort is necessary for the development of stable framework structures, making it cheap and easy to synthesize, for the sustainable application of these broad-spectrum adsorbent materials in water remediation. Therefore, the key new insight of this work was focused on design and synthesis of adsorbent material by employing a new approach involving in-situ mineralization of iron ions in the chitosan hydrogel matrix. This approach offered a sole morphological feature as well as the structural order of the magnetic chitosan polymeric hydrogel beads enabling a noninvasive, fast, environmentally friendly, and low cost separation technique (ca. EUR 2.05 per neodymium magnet disc of 20 mm diameter × 3 mm high) when an external magnetic field is applied during wastewater treatment [6,27]. To promote desorption of organic dye pollutants and adsorbent regeneration, chitosan magnetic gel beads must be properly treated with organic solvents instead of acidic solutions since an acid medium, at very low pH, can dissolve magnetic nanoparticles [25].

The primary objective of this work was the development of a chitosan polysaccharide-based magnetic gel able to remove the Direct Red 83:1 dye from waters, placing special emphasis both on its absorption capacity and the following mechanisms. In order to accomplish this, researchers analyzed experimental data collected at varying dye concentrations and contact times while keeping other parameters constant (such as adsorbent concentration, pH, and temperature). They then applied different kinetic and isotherm models to gain insights into the adsorption behavior of magnetic polymers. To improve the specific surface area and achieve easy separation, chitosan gel beads were synthesized in the presence of Fe nanoparticles, and hydroxyl and/or amine groups at five available sites in the skeleton of chitosan to achieve effective coordination to metal centers. Additionally, a comprehensive characterization of the adsorbent material by FE-SEM, EA, DSC, TGA, IR, and XRD was carried out. This research aims to contribute to the understanding and improvement of wastewater treatment processes using magnetic adsorbents.

## 2. Results and Discussion

### 2.1. Effect of Contact Time

**Figure 1:**
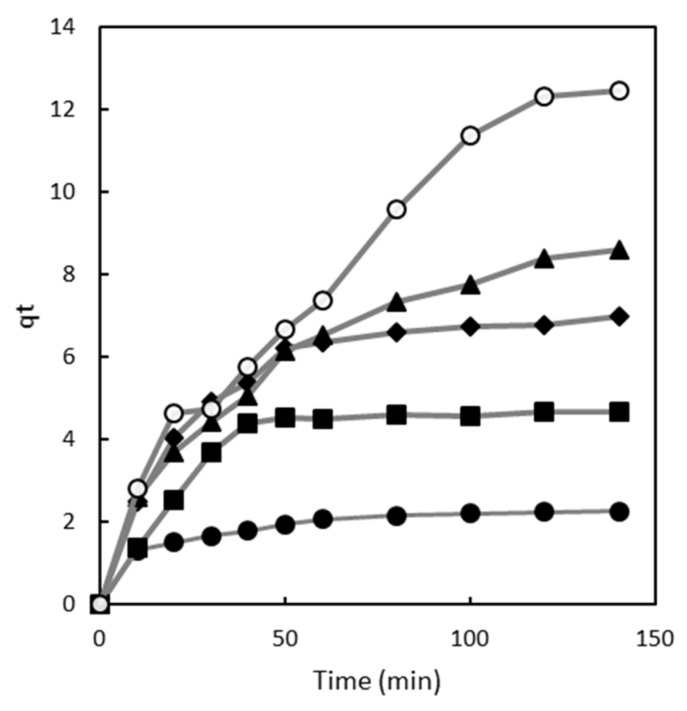
Influence of contact time on chitosan’s magnetic polymer adsorption capacity at various dye concentrations: 50 mg/L (•), 100 mg/L (■), 150 mg/L (♦), 200 mg/L (▲), and 300 mg/L (○).

The adsorption capacity data for various contact times are depicted in Figure 1. The impact of contact time on the adsorption of Direct Red 83:1 was examined at different dye concentrations, from 50 to 300 mg/L. All experiments were carried out at a constant pH of 7.0, using 1 g of the polymer, and with continuous stirring at 500 rpm at room temperature.

Figure 1 shows that the adsorption capacity increased steadily until reaching equilibrium at each dye concentration. At equilibrium, a dynamic balance exists between the adsorbed and desorbed dye within and outside the polymer. The time required to reach this equilibrium is referred to as the equilibrium time, and the amount of dye removed by the polymer at this point represents the maximum adsorption capacity [28].

This behavior can be attributed to the availability of adsorption sites on the polymer surface. At lower dye concentrations (50 mg/L), there are sufficient adsorption sites for dye molecules to rapidly bind, resulting in a shorter equilibrium time. However, as the dye concentration increases (100 mg/L), the available adsorption sites on the polymer surface may begin to saturate. This means that dye molecules must compete for the remaining adsorption sites, which can slow down the adsorption process and consequently increase the time required to reach equilibrium [29,30].

Furthermore, as the dye concentration increases to 150 mg/L and beyond, the adsorption process becomes asymptotic after approximately 60 min of contact time. This suggests that at these higher concentrations, most of the adsorption sites on the polymer surface are already occupied, leading to further slowdown in the adsorption process as the remaining dye molecules vie for the limited available sites [29,30].

However, at higher dye concentrations (200 and 300 mg/L), the adsorption curves did not exhibit the typical asymptotic shape. At 300 mg/L, q_t_ approached 13 mg/g. Instead, the equilibrium time increased with rising concentrations of Direct Red 83:1. These observations provide valuable insights into the adsorption process dynamics and the differing behavior of the adsorbent at various dye concentrations.

### 2.2. Adsorption Kinetics

To ascertain the time required to achieve maximum adsorption and understand the rate-determining step in the adsorption process, we investigated both the time effect and kinetics for the adsorption of Direct Red dye onto the prepared adsorbents. To achieve this, we employed three kinetic models in this study: the pseudo first order, pseudo second order, and intraparticle diffusion model.

#### 2.2.1. Pseudo First Order Model

The objective was to examine the adsorption kinetics and mechanisms involved in the dye’s adsorption onto chitosan magnetic polymers. To this end, the adsorption kinetic data of Direct Red 83:1 were analyzed using three scientific models: the pseudo-first-order model, the pseudo-second-order model, and the intra-particle diffusion model. The linear determination coefficient (*R*^2^) was used as the criterion for identifying the most suitable model.

**Figure 2:**
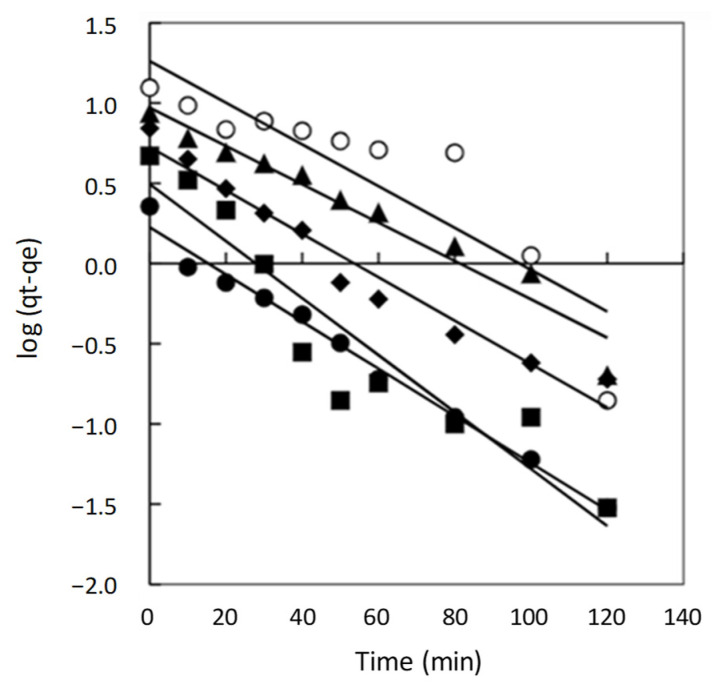
Pseudo first order model plots for chitosan magnetic polymer at different dye concentrations 50 mg/L (•), 100 mg/L (■), 150 mg/L (♦), 200 mg/L (▲), and 300 mg/L (○).

**Figure 3:**
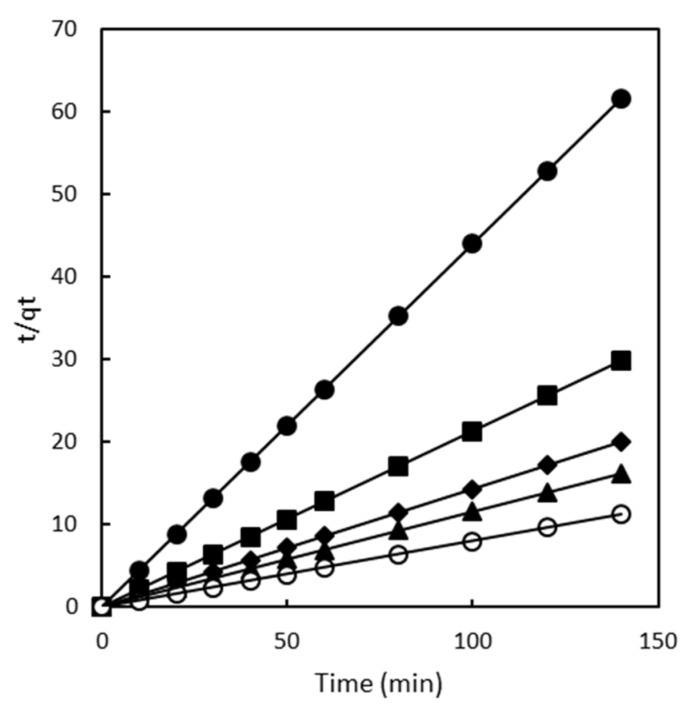
Pseudo second order model plots for chitosan magnetic polymer at different dye concentrations 50 mg/L (•), 100 mg/L (■), 150 mg/L (♦), 200 mg/L (▲), and 300 mg/L (○).

**Figure 4:**
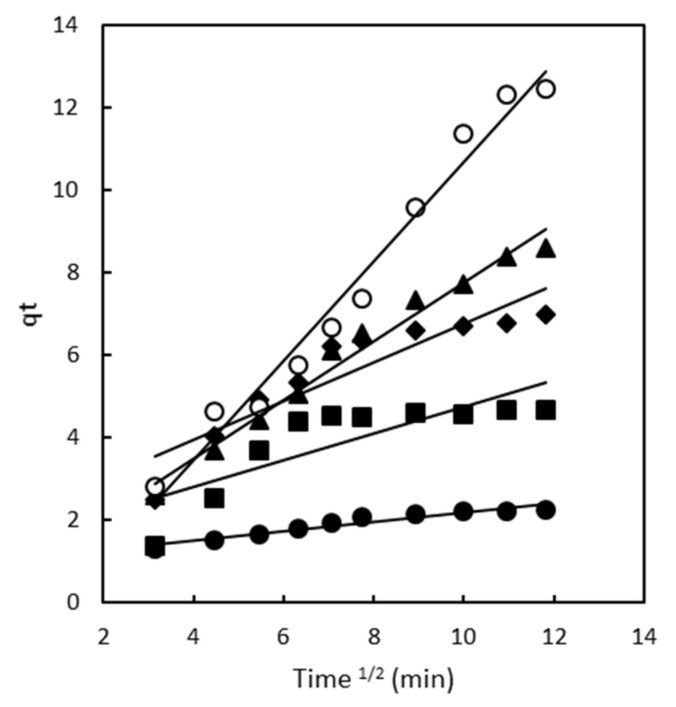
Intraparticle diffusion model plots for chitosan magnetic polymer at different dye concentrations 50 mg/L (•), 100 mg/L (■), 150 mg/L (♦), 200 mg/L (▲), and 300 mg/L (○).

The plots for the pseudo-first-order, pseudo-second-order, and intra-particle diffusion models for the adsorption of Direct Red 83:1 onto the chitosan magnetic adsorbent are presented in Figure 2, Figure 3, and Figure 4, respectively. These plots showcase the fitting of the experimental data to the respective kinetic models, enabling a visual assessment of their agreement with the observed adsorption behavior.

The results and corresponding kinetic parameters, as well as the correlation coefficients (R^2^), are reported in Table S1 (Supplementary Materials). By comparing the R^2^ values for each model, the best-fitting model can be identified, providing valuable insights into the dominant adsorption mechanisms and the overall kinetics of the adsorption process for Direct Red 83:1 onto the chitosan magnetic adsorbent.

#### 2.2.2. Pseudo Second Order Model

As shown in Figure 2, the plot of t/qt versus t resulted in straight lines throughout the entire range of measurement. However, despite this linearity, the R^2^ values obtained from fitting the experimental data to the pseudo-first-order model varied, and the calculated q_e_ (equilibrium adsorption capacity) values were notably different from the experimental q_e_ values. As a consequence, the pseudo-second-order kinetic model emerged as the most suitable model to describe the adsorption kinetics of the dye onto the adsorbent surface.

The application of the pseudo-second-order model indicates that the adsorption process is primarily governed by chemisorption or chemical adsorption, and it is the rate-limiting step that controls the overall adsorption process. This finding aligns with the observation of similar kinetics in various other studies involving different adsorbent systems, such as the adsorption of Malachite Green using magnetic-cyclodextrin-graphene oxide nanocomposites [31], the adsorption of methyl orange and Pb (II) on β-CD and polyethyleneimine bi-functionalized magnetic nano-adsorbent [32], the removal of Eu (III) onto Fe_3_O_4_ cyclodextrin magnetic composite [33], and the removal of organic pollutants on magnetic β-cyclodextrin porous polymer nano-spheres [34].

These collective findings provide strong support for the pseudo-second-order kinetic model as a reliable and consistent approach to understand and characterize the adsorption kinetics of different adsorbent systems for various pollutants.

The adsorption process comprises multiple steps. It initiates with the solute molecules (dye) being transported from the aqueous solution to the surface of the adsorbent (magnetic polymeric gel). Subsequently, the dye molecules diffuse onto the adsorbent, resulting in their adsorption. To comprehensively understand the adsorption of Direct Red 83:1 onto magnetic polymers, researchers analyzed the kinetics using the intraparticle diffusion model. This analysis aims to determine whether intraparticle diffusion significantly influences the overall adsorption process.

#### 2.2.3. Intraparticle Diffusion Model

The intraparticle diffusion model provides insights into the role of intraparticle diffusion in governing the adsorption process. By plotting the amount of Direct Red 83:1 dye absorbed against the square root of time (Figure 4), the influence of intraparticle diffusion on the adsorption kinetics can be assessed. This approach follows the work of Weber in 1963 [35] and Crini in 2007 [36], who employed this model to study similar adsorption phenomena. Analyzing the adsorption process using the intraparticle diffusion model allows researchers to ascertain the contribution of intraparticle diffusion and its significance in determining the rate of the adsorption process. Understanding this aspect is vital in comprehending the overall kinetics and mechanisms of adsorption of Direct Red 83:1 onto the magnetic polymers.

Figure 4 illustrates the plot of qt (amount of Direct Red 83:1 dye adsorbed) versus t^0.5^ (square root of time) for the intraparticle diffusion of Direct Red 83:1 onto chitosan magnetic polymeric gel at different concentrations of the dye. For the chitosan magnetic polymer, a single linear portion was observed for all concentrations studied, indicating that the adsorption process within the polymer was controlled by the diffusion of said molecules to the surface of the polymer. This way of behaving of the polymer corresponds to that observed in the adsorption of Direct Blue 78 onto chitosan-NaOH polymer made by Murcia-Salvador [37]; in his case, his polymer was not magnetic, but carrying out intra-particle diffusion indicate that the adsorption behaviour and kinetics of these dye-polymer systems share common characteristics. However, in the magnetic polymer made with β-CD-EPI-magnetic, it behaves differently, which is observed in this case two distinct regions were observed at high concentrations (200 and 300 mg/L), while a single linear portion was seen at low concentrations (50, 100, and 150 mg/L) [6].

The findings from these studies provide valuable insights into the effectiveness of CDs-EPI and chitosan-NaOH polymers as potential adsorbents for the removal of Direct Blue 78 and Direct Red dye from aqueous solutions [6,28,37]. The consistency of the results across multiple studies strengthens the understanding of the adsorption processes and highlights the potential applicability of these adsorbents in water treatment and wastewater remediation [38].

At low concentrations, the linear portion of the adsorption process within the polymer indicates that diffusion of dye molecules to the polymer surface predominantly governs the process. This observation suggests that intraparticle diffusion is the rate-limiting step for adsorption under these lower concentration conditions [37].

Therefore, intraparticle diffusion is independent of the presence of magnetite and is linked to the type of polymer.

The summary of kinetic parameters obtained from these intraparticle diffusion plots is presented in Table S1 (Supplementary Materials), providing valuable insights into the different stages and mechanisms involved in the adsorption process of Direct Red 83:1 onto the chitosan magnetic polymer at various dye concentrations.

### 2.3. Adsorption Isotherms

The adsorption isotherm plays a crucial role in characterizing the interaction between the dye and the adsorbent, offering valuable information about the adsorption capacity of the adsorbent [39].

#### 2.3.1. Freundlich Isotherm

The Freundlich isotherm applies to both monolayer (chemisorption) and multilayer adsorption (physisorption) processes. It assumes that the adsorbate binds to the heterogeneous surface of the adsorbent. The Freundlich isotherm provides two crucial constants: (K_F_) (the Freundlich coefficient) and (n_F_) (the Freundlich constant) [40]. Typically, favorable adsorption falls within a range of 1 to 10 for (n_F_). A higher (n_F_) value (equivalently, a smaller value of (1/n_F_) indicates a stronger and more effective interaction between the adsorbent and the adsorbate. When (1/n_F_) is less than 1, it corresponds to a normal L-type isotherm, while (1/n_F_) greater than 1 indicates cooperative sorption.

According to the results presented in Table 1 and Figure S1 (Supplementary Materials), the K_F_ value for the magnetic chitosan polymer was found to be 1.28. The most crucial parameter related to this isotherm is n_F_, which represents the heterogeneity factor [41]. In the case of the chitosan magnetic polymer, the value of n_F_ was determined to be 1.68, falling within the favorable adsorption range of 1 to 10. This suggests that the adsorption process is favored and that there is an effective interaction between the adsorbent and the adsorbate [41]. The experimental data were fitted to the Freundlich model, resulting in a determination coefficient of 0.844 for the chitosan polymer. This coefficient represents the goodness of fit of the experimental data to the Freundlich isotherm model for the adsorption process.

#### 2.3.2. Langmuir Isotherm

The Langmuir adsorption isotherm is based on the assumption of homogeneous adsorption, where adsorption occurs in a monolayer coverage on the adsorbent surface. This isotherm assumes that all adsorption sites are equivalent, leading to uniform surface coverage. According to the Langmuir model, the ability of a molecule to adsorb at a specific site is independent of the occupation of neighboring sites.

For the Langmuir isotherm, it is essential to analyze the q_max_ value, which represents the maximum adsorption capacity of the adsorbent under specific experimental conditions. In the case of the magnetic chitosan hydrogel, the q_max_ value was found to be 17.46 as described in Table S1 and Figure S2 (Supplementary Materials). This value provides valuable information about the adsorption capacity of the adsorbent and the maximum amount of dye that can be adsorbed per unit weight of the adsorbent.

#### 2.3.3. Temkin Isotherm

**Figure 5:**
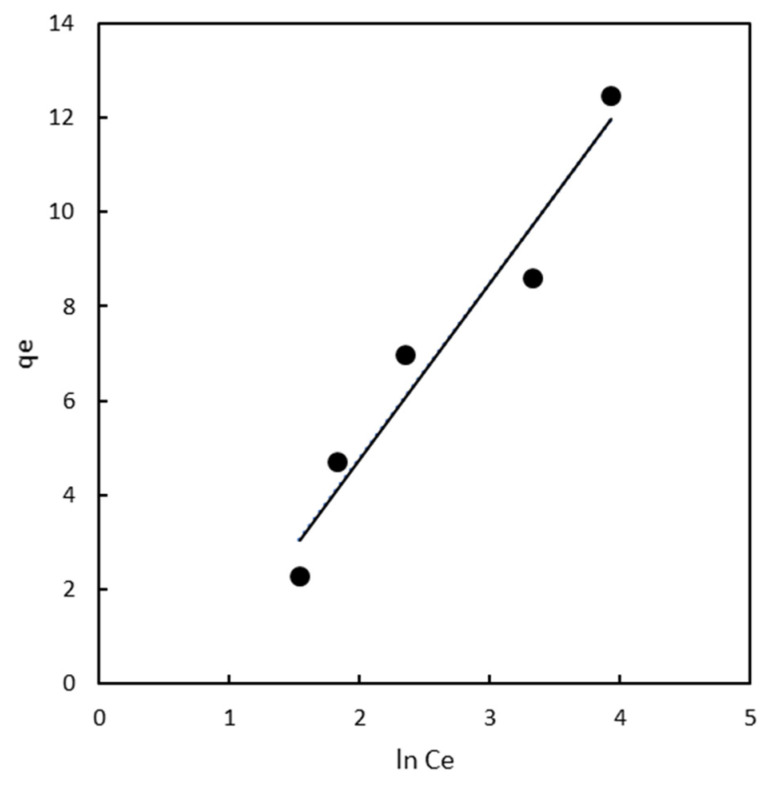
Temkin isotherm plots for chitosan magnetic polymers.

In the current investigation, researchers analysed experimental data by employing the Temkin isotherm. The best fit for the magnetic chitosan polymer was achieved using this model, with a determination coefficient of 0.946, indicating that adsorption occurred on heterogeneous surfaces [40]. The Temkin isotherm model assumes that the heat of adsorption (a function of temperature) for all molecules in the layer decreases linearly rather than logarithmically with coverage (as shown in Figure 5). This model primarily describes the chemical adsorption process as electrostatic interaction. Overall, the Temkin isotherm aligned well with the observed results, highlighting electrostatic interaction as an important mechanism influencing the interaction between the chitosan adsorbent and the pollutant [42].

### 2.4. Chitosan Magnetic Polymer Characterization

#### 2.4.1. Thermogravimetric Analysis

**Figure 6:**
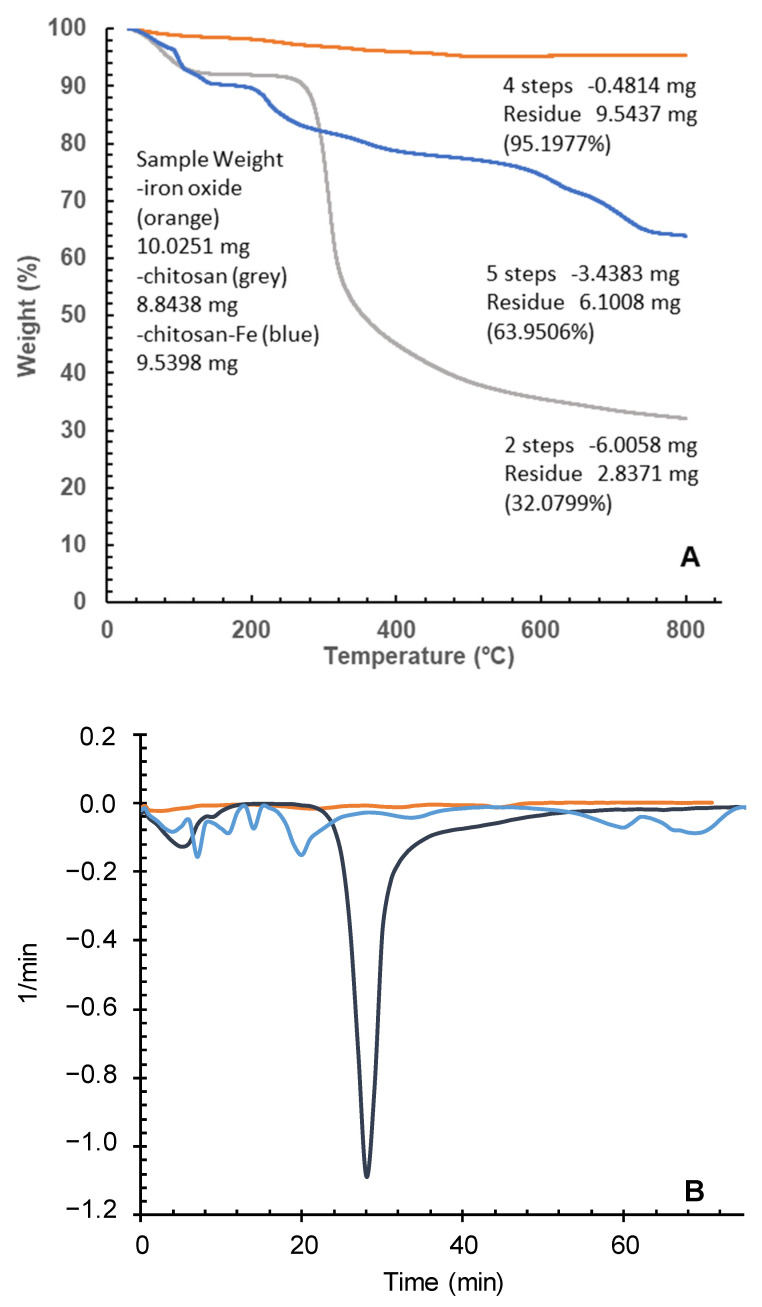
(**A**) TGA of Chitosan-Fe polymeric gel (Blue line), chitosan (Grey Line) and Fe (Orange line). (**B**). DTG of Chitosan-Fe polymeric gel (Blue line), chitosan (Black Line), and Fe (Orange line).

Different patterns of decomposition were obtained for the original chitosan and the iron oxide coated chitosan polymeric gel (Figure 6). Together with a first smooth and sustained weight loss around 70 °C, associated with loss of water, chitosan gel started to degrade at 270 °C due to breakdown of polysaccharide side chains, giving a marked weight loss (40%) in an interval of just 50 °C (from 270 °C to 320 °C). Common elimination of water and volatile compounds at similar lower temperatures was also recorded for the chitosan magnetic polymeric gel, followed by a degradation profile in four not completely defined thermal stages, starting at 200 °C with a weight percentage of 90%, which gradually decreases until reaching 72% at 640 °C. This lower weight loss (close to 18%) in a wide temperature range (440 °C) suggested that the presence of strong interaction of iron particles with the chitosan gel matrix led to a decreased mobility of the polysaccharide side chains. Starting from 640 °C, we identify a second stage of weight loss for the chitosan magnetic polymeric gel with lower weight loss percentage (around 7%) than that of first stage of degradation, with a total mass loss of 36.05%, revealing enlarged quantity of polysaccharide chains in the chitosan magnetic polymeric gel.

No defined major compounds or stable intermediates were identified within the MS fragments, with multiple masses between 30 and 73 amu probably corresponding to the decomposition series of oxygen and nitrogen single bonded compounds C_n_ H_2n+1_ O and C_n_ H_2n+2_ N.

The major weight loss in chitosan gel above 270 °C degrees (exothermic effect) corresponds to the first stage of pyrolysis, when dehydration, depolymerization, and decomposition of the acetylated and deacetylated units of the polysaccharide chains, where most of the organic matter is lost, was not observed in the iron containing chitosan polymer. However, the second and third losses, occurring between 150 and 400 degrees, are very similar to those suffered by the iron particles although to a higher extent (8.42 and 4.09% in the chitosan magnetic polymeric gel (Chitosan-Fe) and 1.56 and 0.85% in the iron particles).

A last significant loss of 7.65% by weight above 700 degrees was registered, leaving a total mass loss almost halved (from 67.91% to 36.05%) than original chitosan with about 64% of matter remaining after the constant heating up to 800 °C (Figure 6B).

From thermogravimetry, the weight percentage of chitosan in the Chitosan-Fe polymeric gel can be calculated as follows Equation (1) [43]:

Chitosan content = Ws (%) − Wn (%)(1)

where the Ws was the weight loss (%) from 120 to 800 °C for the Chitosan-Fe samples, and Wn was the weight loss (%) from 120 to 800 °C for the pure Fe_3_O_4_ nanoparticles. After calculation, the weight percentage of chitosan in Chitosan-Fe reached 31.5%. In the case of Jaafari’s Chitosan-Fe nanoparticles [40], they obtained 16.3%. The double content in organic matter achieved for the in situ generation of iron particles could suggest a higher efficiency in the incorporation into the structure of the chitosan polymeric gel.

#### 2.4.2. DRX Powder X-ray Diffraction

**Figure 7:**
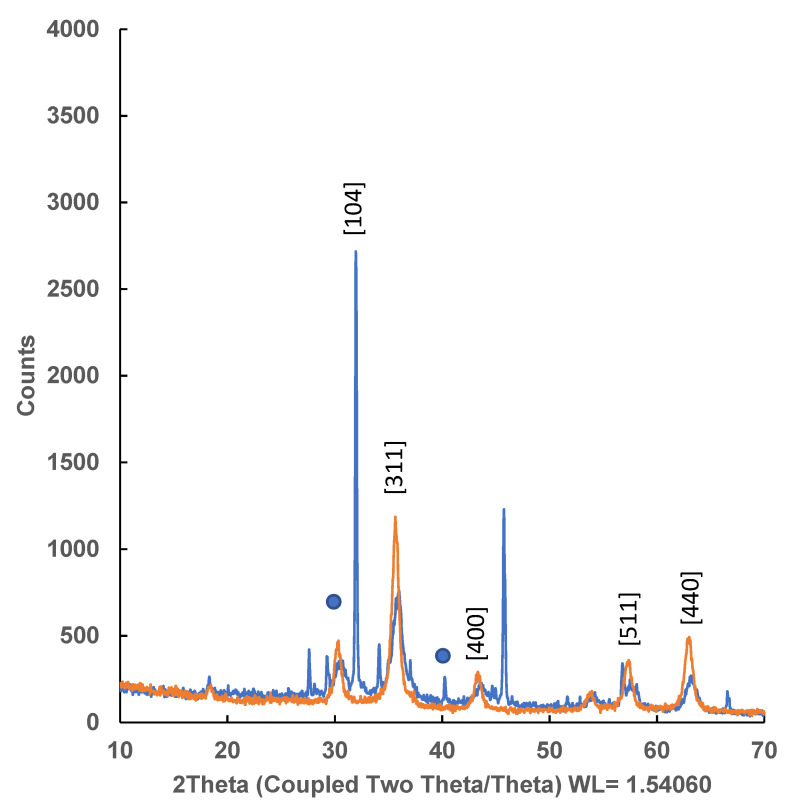
DRX powder X-ray Diffraction of Chitosan-Fe polymeric gel (Blue line) and Fe (Orange line). Blue dot represents non-characteristic magnetite diffractions.

A degree of crystallinity between 68–70% was recorded in the material for the independently prepared iron particles (PI) and for the chitosan magnetic polymeric gel formed in the presence of iron particles. The characteristic face-centred cubic lattice Fd-3m of magnetite (ICDD database, standard sample PDF 04-006-6551 [44]), observed for independent PI particles’ diffraction patterns, is primarily found in the final chitosan iron polymer (Figure 7).

However, novel diffractions were also observed together with those of magnetite, which could be attributed to a distortion of the crystalline structure or to a substitution of magnetite by haematite in some lattices. This last process has been previously described by a partial oxidative substitution of Fe^2+^ or through a redox-independent dissolution-reprecipitation reaction in the acidic media in which they are prepared [45].

#### 2.4.3. FT-IR

**Figure 8:**
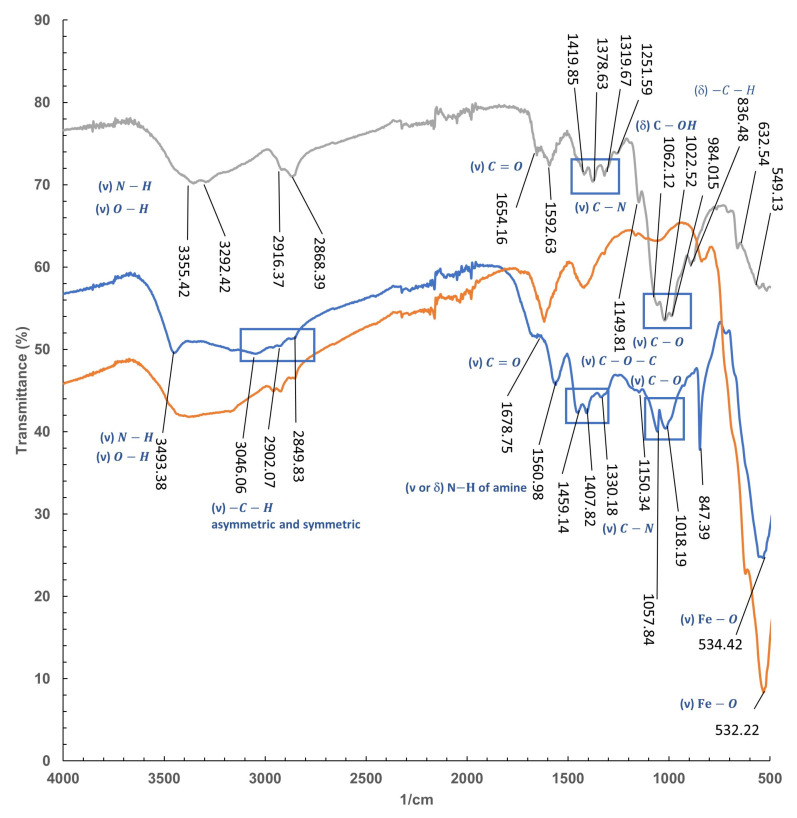
IRTF of Chitosan (Grey line), Chitosan-Fe polymeric gel (Blue line), and Fe (Orange Line).

Similar profiles were observed in the spectrum of commercial chitosan and iron containing chitosan polymer (Figure 8).

Two remarkable peaks at 3355 and 3292 cm^−1^ within a broad band corresponding to N–H and O-H stretching in chitosan are in good agreement with those previously described in literature [46]. These bands seem to be sharpening at a higher frequency 3493 cm^−1^ in the chitosan-based polymer, which could be indicative of losing interactions by hydrogen bonding [47] of at least one of them.

Indeed the absorption bands at 2916 and 2868 cm^−1^ in chitosan attributed to C-H symmetric and asymmetric stretching in CH_2_OH [48], respectively, were also recorded for the polymer with a lower definition.

The stretching vibration corresponding to the double bound C=O (1654 cm^−1^) with less intensity than the N–H bending (1592 cm^−1^), typical for chitosan and related polysaccharides [49], was also found for both compounds. However, significant displacement of this band was observed for the polymer.

The shift of the bands in the case of C=O, from 1654 cm^−1^ to 1678 cm^−1^ in the polymer together with the displacement of the C-N stretching vibrations, originally located at 1419 cm^−1^, 1378 cm^−1^, and 1319 cm^−1^ to higher frequencies (1459, 1407, and 1330 cm^−1^, respectively) could be evidence of an increase in the strain of the structure when forming the coating for the iron particles. Minor displacement was found for the already identified C-O stretching bands of chitosan at 1149 cm^−1^ for the C-O-C bridge, and 1062, 1022, and 948 cm^−1^ [50], which were found in the polymer at frequencies 1150 cm^−1^, and 1057 and 1018 cm^−1^, with the third C-O band not defined, differing around 12 cm^−1^ for the highest one.

Finally, the more relevant band of the iron nanoparticles corresponding to the vibration of the iron-oxygen bonds placed at around 570 cm^−1^ [51] was also identified at 532 cm^−1^ for the nanoparticles used as comparison and almost unaltered at 534 cm^−1^ in the spectrum of the polymer.

#### 2.4.4. FESEM and EDX

**Figure 9:**
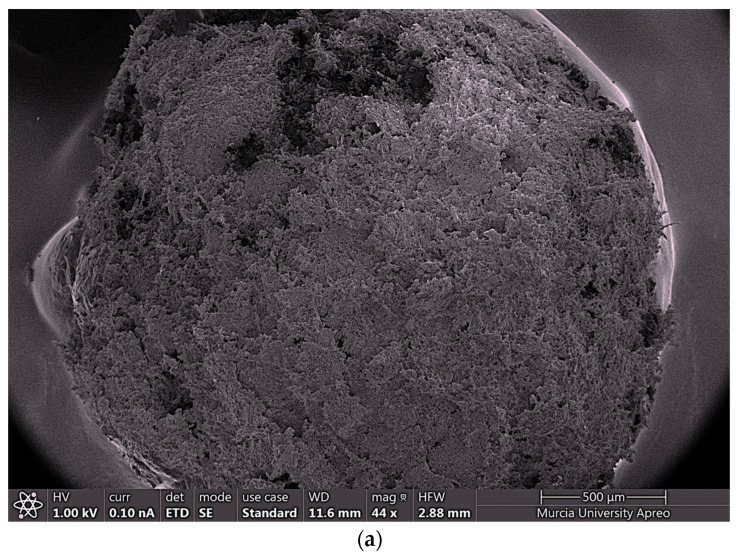

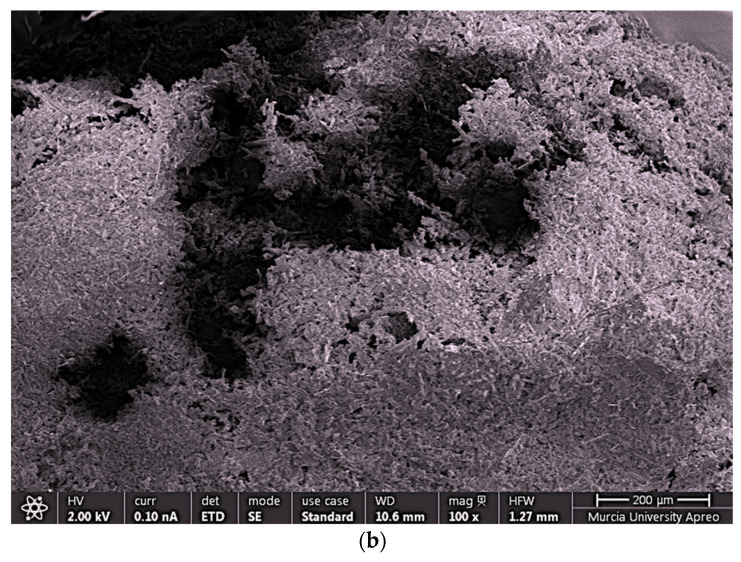
(**a**) Chitosan Polymer. (**b**) Detail of small dark areas of the chitosan polymer.

Several representative polymeric macrostructures were analyzed by field emission scanning electron microscopy to characterize the morphology of iron coated chitosan polymeric hydrogel. Highly regular spherical structures were observed for the polymeric hydrogel (Figure 9a). However, some small dark areas dotted the structure, suggesting the presence of zones where the coating of iron nanoparticles seems to be not completed (Figure 9b).

As can be seen in Figure S3 (Supplementary Materials), the energy-dispersive X-ray spectroscopy (EDX) analysis revealed a huge difference in iron content for both Chitosan (a) and Chitosan-Fe polymeric gel (b), being virtually absent in clear areas and the majority in dark ones, and thus supporting the previous assumption.

#### 2.4.5. Molecular Models

**Figure 10:**
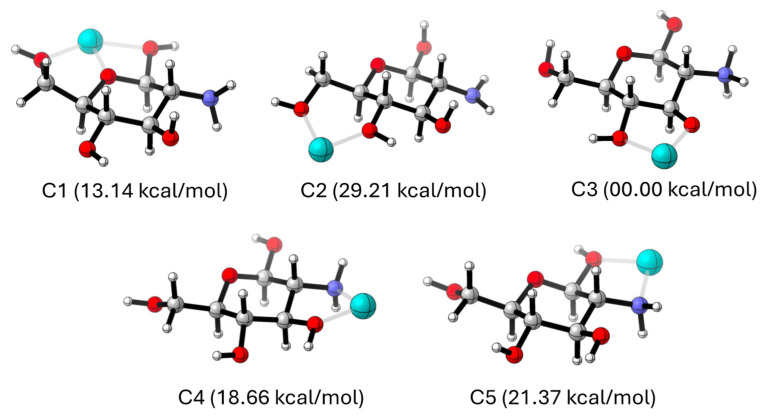
Optimized Chitosan-Fe(II) polymeric gel model systems. Colour atom code: grey: carbon, red: oxygen, blue: nitrogen, cyan: iron.

The interaction of chitosan with iron was eventually assessed by using density functional theory (DFT). Simulations were performed with divalent cations and glucosamine monomers, a model system by Gomes et al. that correctly mimics the stability of chitosan complexes with metallic ions [52]. As discussed by these authors, metal centres can be coordinated to chitosan by hydroxyl and/or amino groups at five available sites (labelled as C1–C5). Figure 10 illustrates the optimized complexes with metals at these positions. Values in parenthesis stand for the computed relative energies in kcal/mol, where the global minimum is taken as reference.

As noted, the C3 coordination leads to the most stable complex, while all other coordination patterns are associated to less favourable (more positive) relative energies, which lie in the range of 13.14–29.21 kcal/mol. Indeed, C3 outperforms both the anchoring to the amino groups of the chitosan entity (C4 and C5) and the alternative C1 and C2 sites, even though the former established three interactions (two hydroxyl contacts + the heterocyclic oxygen). A close inspection of Figure 10 reveals that the large stability at C3 arises from a concomitant proton transfer equilibrium from the hydroxyl to the amino group. Consequently, theory predicts that iron coordination induces a protonation transfer that leads to a deprotonated hydroxyl, which in turn enhances the coordination. This computational outcome agrees the recorded IR spectrum, where Fe–O bands are observed while Fe–N peaks were not present.

### 2.5. Engineering and Economic Feasibilities

The engineering and economic feasibilities of the synthesized chitosan-based magnetic gel beads for dye removal was assessed based on their performance in various experimental setups. The synthesis process of the magnetic gel beads involves straightforward steps, including chitosan dissolution, Fe nanoparticle incorporation, and gel formation. These steps can be easily scaled up for industrial applications. Key engineering aspects include:

- Simplicity of Synthesis: The co-precipitation method used for incorporating Fe nanoparticles into the chitosan gel is simple and cost-effective, making it suitable for large-scale production.
- Operational Conditions: The adsorption process is effective under a wide range of pH and temperature conditions, which aligns well with the varying conditions of industrial wastewater streams.
- Regeneration and Reusability: The magnetic gel beads can be regenerated using organic solvents, enabling multiple cycles of adsorption and desorption without significant loss of efficiency. This reduces the overall operational costs and enhances the sustainability of the process.
- Ease of Separation: The magnetic properties of the gel beads facilitate easy separation from treated water using an external magnetic field. This non-invasive separation technique simplifies the operational procedure and minimizes the need for complex filtration systems.

## 3. Conclusions

The Chitosan-Fe polymeric hydrogel demonstrates a highly efficient removal process for Direct Red 83:1 dye from aqueous solutions, confirming its potential as an effective adsorbent material for wastewater treatment. The adsorption kinetics of the hydrogel align well with the pseudo-second-order kinetic model, exhibiting a high determination coefficient (R^2^ = 0.999) and indicating that a chemisorption mechanism predominantly governs the adsorption process.

Equilibrium adsorption studies revealed that the adsorption behavior is best described by the Temkin isotherm model, with a maximum adsorption capacity (q_max_) of 17.46 mg/g. This finding underscores the hydrogel’s capacity to handle significant dye concentrations, making it suitable for large-scale industrial applications. The magnetic properties of the hydrogel further enhance its practical utility, allowing for easy separation from aqueous solutions via simple magnetic decantation, thus simplifying the post-treatment process and reducing operational costs.

Comprehensive characterization of the Chitosan-Fe hydrogel using thermogravimetry, differential scanning calorimetry, Fourier transform infrared spectrometry, and powder X-ray diffraction analyses confirmed the successful synthesis and robust structural integrity of the hydrogel. These analyses provided insights into the thermal stability and functional group interactions within the hydrogel matrix, validating its resilience under varying environmental conditions.

Theoretical predictions using density functional theory (DFT) models suggested that C3 coordination results in the most stable hydrogel structure. This theoretical insight aligns with the empirical data, providing a deeper understanding of the molecular interactions within the hydrogel matrix and guiding future modifications to enhance its stability and adsorption efficiency.

Despite the presence of small surface gaps observed via field emission scanning electron microscopy (FE-SEM) and energy-dispersive X-ray spectroscopy (EDX), the hydrogel maintains high adsorption efficiency. These surface gaps, likely due to minor structural irregularities, do not significantly impair the hydrogel’s performance, as evidenced by the consistent adsorption capacity and magnetic properties.

The study’s findings indicate that the Chitosan-Fe hydrogel offers a viable and efficient solution for remediating contaminated aqueous environments, particularly those impacted by dyeing industry activities. Its high adsorption capacity, combined with easy magnetic separation, positions it as a competitive alternative to conventional adsorbents, which often suffer from limitations such as difficult recovery and lower adsorption efficiencies.

## 4. Materials and Methods

### 4.1. Chemicals and Reagents

Chitosan, iron (III) chloride hexahydrate (FeCl_3_·6H_2_O), ammonium hydroxide, and ethanol were procured from Sigma-Aldrich (Madrid, Spain). Meanwhile, iron (II) chloride tetrahydrate (FeCl_2_·4H_2_O) was obtained from Fluka (Madrid, Spain). The Direct Red 83:1 dye used in the experiments was supplied by AITEX (Asociación de Investigación de la Industria Textil, Alcoy, Spain).

### 4.2. Chitosan-Fe Polymer Preparation

To prepare the chitosan magnetic beads, the following procedure was followed. 5 g of chitosan powder was dissolved in 250 mL of a 5% acetic acid aqueous solution. The mixture was stirred for 2 h at 50 °C to obtain a homogeneous gel. After achieving a uniform gel, 16 g of FeCl_2_·4H_2_O and 32 g of FeCl_3_·6H_2_O were added to the gel [6]. The mixture was further stirred for 1 h at 50 °C to facilitate the incorporation of iron ions into the gel. To form the polymer beads, the homogeneous gel containing chitosan and iron ions was carefully dropped into a bath containing a 30% aqueous solution of NaOH. Upon contact with the NaOH solution, the gel rapidly transformed into spherical particles, thus forming the chitosan magnetic gel beads (Figure S4, Supplementary Materials). The newly formed beads were then washed with water to remove any residual chemicals or impurities. Finally, the chitosan magnetic polymeric gel beads were left to dry at room temperature, resulting in the final product, ready for use in the experiments (Figure S4, Supplementary Materials).

### 4.3. Dye Solution Preparation

To conduct the adsorption experiments, various concentrations of Direct Red 83:1 were prepared. Direct Red 83:1 is an azo dye with the chemical formula C_33_H_20_N_6_Na_4_O_17_S_4_ and a molecular weight of 992.77 g/mol.

The concentrations of Direct Red 83:1 used in the adsorption tests for both adsorbents were as follows: 50 mg/L, 100 mg/L, 150 mg/L, 200 mg/L, and 300 mg/L. These different concentrations were employed to investigate and evaluate the adsorption properties of the respective adsorbents under varying dye concentrations. By analyzing the adsorption behavior at different dye concentrations, a comprehensive understanding of the adsorption process and efficiency can be obtained.

### 4.4. Analyses and Data Evaluation

The measurements were taken after reaching the equilibrium state between the dye and the adsorbents. The absorbance of the dye solution was measured both before and after the treatment with the polymers at the wavelength of maximum absorbance for the dye, which is λ_max_ = 526 nm. The molar absorptivity (extinction coefficient) of the dye at this wavelength is ε_526_ = 1065 M^−1^cm^−1^. In the adsorption experiments, the concentration of the dye in the supernatant was measured using a spectrophotometer, specifically the Shimadzu UV-1700 model (Kyoto, Japan).

By comparing the absorbance values before and after the treatment, the extent of dye adsorption onto the chitosan magnetic polymer can be determined. This analysis helps in evaluating the effectiveness of the adsorbents in removing the dye from the solution at different concentrations.

### 4.5. Adsorption Experiments

The adsorption tests were conducted at a constant temperature of 25 °C, utilizing solutions containing various concentrations of the dye ranging from 50 to 300 mg/L (Figure S5, Supplementary Materials). For each experiment, 1 g of the chitosan magnetic polymeric gel was mixed with 50 mL of the dye solution. The mixture was continuously stirred at a fixed speed of 500 rpm throughout the experiment to ensure uniform contact between the dye and the adsorbent.

To determine the amount of dye that was not retained by the magnetic polymer at specific time intervals (every 10 min), external ferrite magnets were employed. The mixture was subjected to the influence of these magnets for 5 min, facilitating the separation of the magnetic polymer beads along with the adsorbed dye from the solution (Figure S5, Supplementary Materials).

Once the separation was accomplished, the concentration of the remaining dye in the solution was measured using the spectrophotometer, which allows for precise and accurate quantification of the dye’s concentration. This procedure was repeated for each concentration of dye tested, and the data obtained from the spectrophotometer measurements were used to assess the adsorption efficiency of the polymer under varying dye concentrations and contact times.

The amount of dye adsorbed on the polymer (q_t_) in mg/g was determined using a mass balance approach, as described by Renard et al. in 1997 [53]. The equation used for this determination is given by Equation (2) [53]:

(2) $$q_{t}=\frac{V(C_{o}-\;C_{t})}{m}$$

where:

- q_t_ is the amount of dye adsorbed on the polymer at time t (mg/g).
- C_o_ is the initial concentration of dye in the solution (mg/L).
- C_t_ is the concentration of dye in the solution at time t (mg/L).
- V is the volume of the dye solution used (L).
- m is the mass of the polymer used (g)

### 4.6. Adsorption Kinetics

The study of kinetics holds significant importance in adsorption research as it allows us to predict the rate at which pollutants are eliminated from aqueous solutions and provides valuable insights into the mechanism of sorption reactions. To gain a deeper understanding of the mechanism behind dye adsorption onto the newly developed adsorbents, several kinetic models, including the pseudo first order, pseudo second order, and intra-particle diffusion models, were carefully examined using the experimental data. These models were utilized to analyze and characterize the adsorption process, enabling a comprehensive investigation of the interactions between the adsorbents and the dye molecules.

The pseudo-first-order rate of Lagergren [15] is given by the Equation (3):

(3) $$\log\left(q_{e}-q_{t}\right)=\log q_{e}-\frac{k_{1}}{2.303}t$$

This model assumes that the rate of adsorption is directly proportional to the difference between the equilibrium adsorption capacity (*q_e_*) and the adsorption capacity at any given time (*q_t_*). By fitting the experimental data to this model, the rate constant (k_1_) and other kinetic parameters can be determined, providing insights into the adsorption mechanism and the rate-limiting step of the process.

The adsorption kinetics can be effectively described by a pseudo-second-order model, as proposed by Ho [54]. This model is represented by Equation (4):

(4) $$\frac{t}{q_{t}}=\frac{1}{{k_{2}q_{e}}^{2\;}}+\frac{1}{q_{e}}t$$

The pseudo-second-order model posits that the adsorption rate is influenced by the interaction between the adsorbate (dye) and the adsorbent (the prepared adsorbents). Unlike the pseudo-first-order model, the pseudo-second-order model considers that the rate of adsorption is directly proportional to the square of the equilibrium adsorption capacity (*q_e_*) and inversely proportional to the adsorption capacity at any given time (*q_t_*).

Fitting the experimental data to this model allows us to determine the rate constant (k_2_) and further understand the adsorption mechanism, providing valuable information about the kinetics of the adsorption process.

The intraparticle diffusion model proposed by Weber and Morris [35] expresses the root time dependence through the following Equation (5):

(5) $$qt=\mathrm{ki}\sqrt{t}+C$$

The intraparticle diffusion model is used to investigate the intraparticle diffusion mechanism during the adsorption process. It suggests that the adsorption occurs in multiple steps, including external surface adsorption, intraparticle diffusion, and equilibrium. The root time dependence in Equation (3) indicates that the rate of intraparticle diffusion is proportional to the square root of the contact time (t^0.5^).

Fitting the experimental data to this model allows us to determine the intraparticle diffusion rate constant (k_i_) and evaluate the contribution of intraparticle diffusion to the overall adsorption process. Additionally, the intercept (C) provides insights into the boundary layer effect, which represents the contribution of external surface adsorption to the overall adsorption mechanism.

### 4.7. Isotherm Analysis

The adsorption isotherm plays a critical role in understanding how adsorbing molecules are distributed between the liquid and solid phases once the adsorption process reaches an equilibrium state. To optimize the design of an adsorption system for efficiently removing Direct Red 83:1 from solutions, it becomes crucial to identify the most suitable correlation for the equilibrium studies. In this study, three different isotherms, namely Freundlich, Langmuir, and Temkin isotherms, were employed and fitted to the experimental data.

By utilizing these isotherms, the adsorption behavior of Direct Red 83:1 onto the adsorbents can be thoroughly investigated, and the appropriate model can be selected based on its best fit to the experimental data. This choice will aid in obtaining valuable information about the adsorption capacity, surface characteristics, and interactions between the adsorbent and the dye molecules, ultimately contributing to the optimal design and performance of the adsorption system.

The linearized form of the Freundlich isotherm model is expressed by the following Equation (6):

(6) $$\ln\mathrm{qe}=\ln K_{F}+\frac{1}{n_{F}}\ln\mathrm{Ce}$$

where *q_e_* is the equilibrium dye concentration on absorbent (mg/g), *C_e_* the equilibrium dye concentration in solution (mg/L), *K_F_* the Freundlich constant (L/g) and 1/*n_F_* is the heterogeneity factor. The plot of ln *q_e_* versus ln*C_e_* was used to determine the intercept value of *K_F_* and the slope of 1/*n_F_*.

By analyzing the experimental data and fitting them to the Freundlich isotherm model, valuable information about the adsorption capacity, intensity, and favorability of the adsorption process can be obtained, providing insights into the interaction between the dye and the adsorbent.

The linearized form of the Langmuir model is given by the following Equation (7):

(7) $$\frac{\mathrm{Ce}}{\mathrm{qe}}=\frac{1}{K_{L}}+\frac{a_{L}}{K_{L}}\mathrm{Ce}\;$$

where *C_e_* (mg/L) and *q_e_* (mg/g) are the liquid phase concentration and solid phase concentration of adsorbate at equilibrium respectively. *K_L_* (L/g) and *a_L_* (L/mg) are the Langmuir isotherm constants. Plotting *C_e_/q_e_* versus *C_e_* makes it possible to know the value of *K_L_* from the intercept (1/*K_L_*) and the value of *a_L_* from the slope (*a_L_/K_L_*); *q_max_* is the maximum adsorption capacity of the polymer and is defined by *K_L_/a_L_*.

The most important characteristic of this isotherm can be described using a dimensionless constant (*R_L_*), which is called separation factor and is described by the following Equation (8):

(8) $$R_{L}=\frac{1}{1+a_{L}C_{o}}$$

The value of the separation factor (*R_L_*) provides insight into the nature of adsorption as follows:

*R_L_* > 1: Indicates unfavorable adsorption, meaning the adsorption capacity decreases with increasing equilibrium concentration. This suggests that the adsorption process is less efficient at higher dye concentrations.

*R_L_* = 1: Indicates linear adsorption, implying a constant adsorption capacity regardless of the equilibrium concentration.

0 < *R_L_* < 1: Indicates favorable adsorption, where the adsorption capacity increases with increasing equilibrium concentration. This suggests that the adsorption process is more efficient at higher dye concentrations.

The separation factor (*R_L_*) is a valuable tool for assessing the feasibility and efficiency of the adsorption process and helps to understand the type of adsorption occurring on the adsorbent surface.

Temkin isotherm is an alternative model that assumes a linear decrease in the heat of sorption as the adsorption process progresses, in contrast to the logarithmic relationship in the Freundlich equation. The Temkin isotherm takes into account the interactions between the adsorbate and the adsorbent, considering the adsorbent’s finite surface area and the heterogeneity of the surface [42].

The Temkin isotherm equation suggests a linear decrease in sorption energy as the adsorption sites on the adsorbent become saturated. This implies that the heat of sorption gradually decreases as the adsorption progresses.

The linearized form of the Temkin isotherm model is expressed by the following Equation (9):

(9) $$\mathrm{qe}=\frac{\mathrm{RT}}{\mathrm{bT}}\ln\mathrm{aT}+\frac{\mathrm{RT}}{\mathrm{bT}}\ln\mathrm{Ce}$$

where *b_T_* is the Temkin constant, related to the heat of adsorption (kJ/mol), *a_T_* is the constant of Temkin isotherm (L/g), *R* the universal gas constant (8.314 J/mol K), and *T* is the absolute temperature in Kelvin.

### 4.8. Chitosan Magnetic Polymer Characterization

Thermogravimetric analysis was conducted using a thermobalance model TGA/DSC 1 HT (Mettler-Toledo GmbH, Schwerzenbach, Switzerland) coupled with a Balzers Thermostar mass spectrometer (Pfeiffer Vacuum, Asslar, Germany) for gas analysis. Scan bar graph cycles were executed in the 15–94 *m*/*z* range using the quadrupole mass spectrometer (QMS 200 M3), with a dwell time of 2 s for each ion and a cathode voltage of 65 V in the ion source.

About 10 mg of sample were used to study the thermal decomposition from 30 to 800 °C at a heating rate of 10 °C/min under N_2_ atmosphere with a flow rate of 50 mL/min. During the experiment, the sample was placed in an uncovered sapphire crucible of 70 μL capacity. The result was corrected with a blank curve. For each sample, the *m/z*-ratio profiles in the range 15–94 amu, the experimental mass loss (TGA curve) and its first derivative (DTG curve) were recorded.

The crystalline phases in pre-formed iron nanoparticles and within the polymer were also analysed. An x-ray powder diffractometer in mode θ-θ (Bruker D8 Advance, Bruker Corporation, Billerica, MA, USA), using Cu Kα radiation at 40 kV and 30 mA and one-dimensional detector with a 2° window. The primary optics consisted of a 2° Soller slit, a 1 mm incidence slit and an air anti-scattering screen. The secondary optics included an 8 mm anti-scattering slit, a Ni filter, and a 2.5° Soller slit.

Approximately 1 g of powder reduced iron coated macrostructures was placed on a back-loaded sample holder and analysed at steps of 0.05° (dwell time of 1 s/step) over an angular range of 10–70° and 30 rpm of angular velocity. The software DIFFRAC.EVA 5.2 (Bruker AXS, 2020, Billerica, MA, USA) was used to assess the powder diffraction file, along with the crystalline powder database PDF-4+ 2021 (ICDD).

The infrared study was performed on a Nicolet 5700 spectrometer (Nicolet, Madison, WI, USA) equipped with a Ge/KBr beam splitter, a DTGS-KBr detector, and a ceramic infrared source. The sampling module was the Smart Orbit diamond attenuated total reflectance attenuator accessory. The samples were analyzed, divided, and pre-dried at 60 °C. Spectral analysis was performed in the range 4000 to 400 cm^−1^, with a resolution of 4 cm^−1^.

FESEM uncovered samples mounted with the aid of conductive carbon cement on aluminum stubs. FESEM-EDX analyses were performed with a FE-SEM (ApreoS Lovac IML Thermofisher, Waltham, MA, USA) and coupled with an Octane Plus EDAX microanalyzer (AMETEK, Warrendale, PA, USA). Sample analyses were performed between 1–2 kV and 20 kV for EDX analysis.

### 4.9. Computational Calculations

All DFT calculations were performed with the Gaussian 16 suite of programs [55]. The molecular structures were fully optimized with the dispersion-corrected version of the B3LYP functional (B3LYP-D3) [56,57]. This functional has been successfully used for simulating chitosan-metal complexes [58]. The Def2SVP basis set was used for all atoms except Fe, which was treated with the relative effective core potentials (Def2-ECP) [59,60]. Frequencies were also computed to confirm that the localized structures correctly correspond to real minima in the potential energy surface.

## Figures and Tables

**Table 1:** Freundlich, Langmuir, and Temkin parameters.

Isotherm	Parameter	Chitosan-Fe
Freundlich	K_F_	1.28
	n_F_	1.68
	R^2^	0.844
Langmuir	q_max_	17.46
	K_L_	0.76
	a_L_	0.043
	R^2^	0.884
	R_L_	0.13–0.024
Temkin	a_T_	0.48
	b_T_	0.67
	R^2^	0.946

## Data Availability

The data presented in this study are openly available in article.

## Supplementary Materials

The following supporting information can be downloaded at: https://www.mdpi.com/article/10.3390/gels10080496/s1, Figure S1 Freundlich isotherm plots for chitosan magnetic adsorbents. Figure S2 (a) Langmuir isotherm plots. (b) Separation factor. Figure S3 (a) EDX of Chitosan and (b) Chitosan-Fe magnetic polymer. Figure S4 Chitosan magnetic polymeric gel beads. (a) before drying process, (b) after drying process. Figure S5 Adsorption tests. (a) Solutions containing 50 mg/L and 200 mg/L of Direct Red 83:1. (b) Separation of the magnetic polymer (1 g) after 10 min of contact time, by influence of an external ferrite magnet for 5 min. Table S1. Kinetic parameters (pseudo 1st, pseudo 2nd and intraparticle diffusion models) for adsorption of Direct Red 83:1 onto chitosan magnetic polymeric gel.

## References

[1] Ahmed J., Thakur A., Goyal A., Shah M.P. (2021). Industrial Wastewater and Its Toxic Effects. Biological Treatment of Industrial Wastewater.

[2] Markandeya, Mohan D., Shukla S.P. (2022). Hazardous Consequences of Textile Mill Effluents on Soil and Their Remediation Approaches. Clean. Eng. Technol..

[3] Azanaw A., Birlie B., Teshome B., Jemberie M. (2022). Textile Effluent Treatment Methods and Eco-Friendly Resolution of Textile Wastewater. Case Stud. Chem. Environ. Eng..

[4] Tara N., Siddiqui S.I., Rathi G., Chaudhry S.A., Inamuddin, Asiri A.M. (2020). Nano-Engineered Adsorbent for the Removal of Dyes from Water: A Review. Curr. Anal. Chem..

[5] Rápó E., Tonk S. (2021). Factors Affecting Synthetic Dye Adsorption; Desorption Studies: A Review of Results from the Last Five Years (2017–2021). Molecules.

[6] Rodríguez-López M.I., Pellicer J.A., Gómez-Morte T., Auñón D., Gómez-López V.M., Yáñez-Gascón M.J., Gil-Izquierdo Á., Cerón-Carrasco J.P., Crini G., Núñez-Delicado E. (2022). Removal of an Azo Dye from Wastewater through the Use of Two Technologies: Magnetic Cyclodextrin Polymers and Pulsed Light. Int. J. Mol. Sci..

[7] Al-Sakkaf B.M., Nasreen S., Ejaz N. (2020). Degradation Pattern of Textile Effluent by Using Bio and Sono Chemical Reactor. J. Chem..

[8] Morin-Crini N., Lichtfouse E., Fourmentin M., Ribeiro A.R.L., Noutsopoulos C., Mapelli F., Fenyvesi É., Vieira M.G.A., Picos-Corrales L.A., Moreno-Piraján J.C. (2022). Removal of Emerging Contaminants from Wastewater Using Advanced Treatments. A Review. Environ. Chem. Lett..

[9] Saheed I.O., Oh W.D., Suah F.B.M. (2021). Chitosan Modifications for Adsorption of Pollutants—A Review. J. Hazard. Mater..

[10] Keshvardoostchokami M., Majidi M., Zamani A., Liu B. (2021). A Review on the Use of Chitosan and Chitosan Derivatives as the Bio-Adsorbents for the Water Treatment: Removal of Nitrogen-Containing Pollutants. Carbohydr. Polym..

[11] Kyzas G.Z., Bikiaris D.N., Mitropoulos A.C. (2017). Chitosan Adsorbents for Dye Removal: A Review: Chitosan Adsorbents for Dye Removal. Polym. Int..

[12] Barreca S., Oliveri I.P., Lo Presti F., Oliveri V., Giannakis S., Tuccitto N., Spampinato V., Auditore A. (2024). An Innovative “Up-and-Down” Adsorption Processes for Pyrene Removal from Acid Wastewater as New Approach in Water Remediation. Sep. Purif. Technol..

[13] Da Silva Alves D.C., Healy B., Pinto L.A.D.A., Cadaval T.R.S., Breslin C.B. (2021). Recent Developments in Chitosan-Based Adsorbents for the Removal of Pollutants from Aqueous Environments. Molecules.

[14] Stanciu M.-C., Teacă C.-A. (2024). Natural Polysaccharide-Based Hydrogels Used for Dye Removal. Gels.

[15] Zhao X., Wang X., Lou T. (2021). Preparation of Fibrous Chitosan/Sodium Alginate Composite Foams for the Adsorption of Cationic and Anionic Dyes. J. Hazard. Mater..

[16] Lakkaboyana S.K., Soontarapa K., Vinaykumar, Marella R.K., Kannan K. (2021). Preparation of Novel Chitosan Polymeric Nanocomposite as an Efficient Material for the Removal of Acid Blue 25 from Aqueous Environment. Int. J. Biol. Macromol..

[17] Cui J., Wang X., Yu S., Zhong C., Wang N., Meng J. (2020). Facile Fabrication of Chitosan-Based Adsorbents for Effective Removal of Cationic and Anionic Dyes from Aqueous Solutions. Int. J. Biol. Macromol..

[18] Raval N.P., Mukherjee S., Shah N.K., Gikas P., Kumar M. (2021). Hexametaphosphate Cross-Linked Chitosan Beads for the Eco-Efficient Removal of Organic Dyes: Tackling Water Quality. J. Environ. Manag..

[19] Yang Z., Yang H., Jiang Z., Cai T., Li H., Li H., Li A., Cheng R. (2013). Flocculation of Both Anionic and Cationic Dyes in Aqueous Solutions by the Amphoteric Grafting Flocculant Carboxymethyl Chitosan-Graft-Polyacrylamide. J. Hazard. Mater..

[20] Mittal H., Al Alili A., Morajkar P.P., Alhassan S.M. (2021). GO Crosslinked Hydrogel Nanocomposites of Chitosan/Carboxymethyl Cellulose—A Versatile Adsorbent for the Treatment of Dyes Contaminated Wastewater. Int. J. Biol. Macromol..

[21] Chanajaree R., Sriuttha M., Lee V.S., Wittayanarakul K. (2021). Thermodynamics and Kinetics of Cationic/Anionic Dyes Adsorption on Cross-Linked Chitosan. J. Mol. Liq..

[22] Jamali M., Akbari A. (2021). Facile Fabrication of Magnetic Chitosan Hydrogel Beads and Modified by Interfacial Polymerization Method and Study of Adsorption of Cationic/Anionic Dyes from Aqueous Solution. J. Environ. Chem. Eng..

[23] Singh N., Riyajuddin S., Ghosh K., Mehta S.K., Dan A. (2019). Chitosan-Graphene Oxide Hydrogels with Embedded Magnetic Iron Oxide Nanoparticles for Dye Removal. ACS Appl. Nano Mater..

[24] Hingrajiya R.D., Patel M.P. (2023). Fe_3_O_4_ Modified Chitosan Based Co-Polymeric Magnetic Composite Hydrogel: Synthesis, Characterization and Evaluation for the Removal of Methylene Blue from Aqueous Solutions. Int. J. Biol. Macromol..

[25] Frachini E., Petri D. (2019). Magneto-Responsive Hydrogels: Preparation, Characterization, Biotechnological and Environmental Applications. J. Braz. Chem. Soc..

[26] Villamin M.E., Kitamoto Y. (2019). Influence of pH on Dynamic Magnetic Susceptibility of Iron-Oxide Nanoparticles in a Chitosan Hydrogel Matrix. IEEE Trans. Magn..

[27] Nithya R., Thirunavukkarasu A., Sathya A.B., Sivashankar R. (2021). Magnetic Materials and Magnetic Separation of Dyes from Aqueous Solutions: A Review. Environ. Chem. Lett..

[28] Pellicer J., Rodríguez-López M., Fortea M., Lucas-Abellán C., Mercader-Ros M., López-Miranda S., Gómez-López V., Semeraro P., Cosma P., Fini P. (2019). Adsorption Properties of β- and Hydroxypropyl-β-Cyclodextrins Cross-Linked with Epichlorohydrin in Aqueous Solution. A Sustainable Recycling Strategy in Textile Dyeing Process. Polymers.

[29] Botleng J., Patel T., Lata R., Chang R., Rohindra D. (2024). Adsorption of Azo Dyes onto Environmentally Friendly Bacterial Cellulose/Kappa-Carrageenan Hydrogel: Isotherm and Kinetic Studies. Adsorption.

[30] Devi S.G., Amalan A.J., Subasini S., Pius A., Tharini J., Thomas S. (2024). Adsorption and Desorption of Dyes. Carbon Nanomaterials and Their Composites as Adsorbents.

[31] Wang D., Liu L., Jiang X., Yu J., Chen X. (2015). Adsorption and Removal of Malachite Green from Aqueous Solution Using Magnetic β-Cyclodextrin-Graphene Oxide Nanocomposites as Adsorbents. Colloids Surf. Physicochem. Eng. Asp..

[32] Chen B., Chen S., Zhao H., Liu Y., Long F., Pan X. (2019). A Versatile β-Cyclodextrin and Polyethyleneimine Bi-Functionalized Magnetic Nanoadsorbent for Simultaneous Capture of Methyl Orange and Pb(II) from Complex Wastewater. Chemosphere.

[33] Guo Z., Li Y., Pan S., Xu J. (2015). Fabrication of Fe_3_O_4_@ Cyclodextrin Magnetic Composite for the High-Efficient Removal of Eu (III). J. Mol. Liq..

[34] Liu D., Huang Z., Li M., Sun P., Yu T., Zhou L. (2019). Novel Porous Magnetic Nanospheres Functionalized by β-Cyclodextrin Polymer and Its Application in Organic Pollutants from Aqueous Solution. Environ. Pollut..

[35] Weber Jr W.J., Morris J.C. (1963). Kinetics of Adsorption on Carbon from Solution. J. Sanit. Eng. Div..

[36] Crini G., Peindy H.N., Gimbert F., Robert C. (2007). Removal of CI Basic Green 4 (Malachite Green) from Aqueous Solutions by Adsorption Using Cyclodextrin-Based Adsorbent: Kinetic and Equilibrium Studies. Sep. Purif. Technol..

[37] Murcia-Salvador A., Pellicer J.A., Fortea M.I., Gómez-López V.M., Rodríguez-López M.I., Núñez-Delicado E., Gabaldón J.A. (2019). Adsorption of Direct Blue 78 Using Chitosan and Cyclodextrins as Adsorbents. Polymers.

[38] Pellicer J.A., Rodríguez-López M.I., Fortea M.I., Gabaldón Hernández J.A., Lucas-Abellán C., Mercader-Ros M.T., Serrano-Martínez A., Núñez-Delicado E., Cosma P., Fini P. (2018). Removing of Direct Red 83:1 Using α- and HP-α-CDs Polymerized with Epichlorohydrin: Kinetic and Equilibrium Studies. Dye. Pigment..

[39] Bensalah H., Younssi S.A., Ouammou M., Gurlo A., Bekheet M.F. (2020). Azo Dye Adsorption on an Industrial Waste-Transformed Hydroxyapatite Adsorbent: Kinetics, Isotherms, Mechanism and Regeneration Studies. J. Environ. Chem. Eng..

[40] Jaafari J., Barzanouni H., Mazloomi S., Amir Abadi Farahani N., Sharafi K., Soleimani P., Haghighat G.A. (2020). Effective Adsorptive Removal of Reactive Dyes by Magnetic Chitosan Nanoparticles: Kinetic, Isothermal Studies and Response Surface Methodology. Int. J. Biol. Macromol..

[41] Gupta A., Sharma V., Mishra P.K., Ekielski A. (2022). A Review on Polyacrylonitrile as an Effective and Economic Constituent of Adsorbents for Wastewater Treatment. Molecules.

[42] Hu Q., Lan R., He L., Liu H., Pei X. (2023). A Critical Review of Adsorption Isotherm Models for Aqueous Contaminants: Curve Characteristics, Site Energy Distribution and Common Controversies. J. Environ. Manag..

[43] Zhang L., Zhu X., Sun H., Chi G., Xu J., Sun Y. (2010). Control Synthesis of Magnetic Fe_3_O_4_–Chitosan Nanoparticles under UV Irradiation in Aqueous System. Curr. Appl. Phys..

[44] International Centre for Diffraction Data (2024). Powder Diffraction FileTM (PDF®), Muestra Estándar PDF 04-006-6551 [Base de Datos].

[45] Otake T., Wesolowski D.J., Anovitz L.M., Allard L.F., Ohmoto H. (2010). Mechanisms of Iron Oxide Transformations in Hydrothermal Systems. Geochim. Cosmochim. Acta.

[46] Fernandes Queiroz M., Melo K., Sabry D., Sassaki G., Rocha H. (2014). Does the Use of Chitosan Contribute to Oxalate Kidney Stone Formation?. Mar. Drugs.

[47] Larkin P. (2018). Infrared and Raman Spectroscopy: Principles and Spectral Interpretation.

[48] Jantzen Da Silva Lucas A., Quadro Oreste E., Leão Gouveia Costa H., Martín López H., Dias Medeiros Saad C., Prentice C. (2021). Extraction, Physicochemical Characterization, and Morphological Properties of Chitin and Chitosan from Cuticles of Edible Insects. Food Chem..

[49] Sánchez L.-F., Cánepa J., Kim S., Nakamatsu J. (2021). A Simple Approach to Produce Tailor-Made Chitosans with Specific Degrees of Acetylation and Molecular Weights. Polymers.

[50] Hong T., Yin J.-Y., Nie S.-P., Xie M.-Y. (2021). Applications of Infrared Spectroscopy in Polysaccharide Structural Analysis: Progress, Challenge and Perspective. Food Chem. X.

[51] Cha Y.-J., Kim M.-J., Choa Y.-H., Kim J., Nam B., Lee J., Kim D.H., Kim K.H. (2010). Synthesis and Characterizations of Surface-Coated Superparamagentic Magnetite Nanoparticles. IEEE Trans. Magn..

[52] Gomes J.R.B., Jorge M., Gomes P. (2014). Interaction of Chitosan and Chitin with Ni, Cu and Zn Ions: A Computational Study. J. Chem. Thermodyn..

[53] Renard P., De Marsily G. (1997). Calculating Equivalent Permeability: A Review. Adv. Water Resour..

[54] Ho Y.-S. (2006). Review of Second-Order Models for Adsorption Systems. J. Hazard. Mater..

[55] Gaussian 16 A03. https://gaussian.com/citation_a03/.

[56] Grimme S. (2011). Density Functional Theory with London Dispersion Corrections. WIREs Comput. Mol. Sci..

[57] Becke A.D. (1993). A New Mixing of Hartree–Fock and Local Density-Functional Theories. J. Chem. Phys..

[58] Ataei S., Nemati-Kande E., Bahrami A. (2023). Quantum DFT Studies on the Drug Delivery of Favipiravir Using Pristine and Functionalized Chitosan Nanoparticles. Sci. Rep..

[59] Weigend F., Ahlrichs R. (2005). Balanced Basis Sets of Split Valence, Triple Zeta Valence and Quadruple Zeta Valence Quality for H to Rn: Design and Assessment of Accuracy. Phys. Chem. Chem. Phys..

[60] Weigend F. (2006). Accurate Coulomb-Fitting Basis Sets for H to Rn. Phys. Chem. Chem. Phys..

