# Supplementary material for: Development of Chitosan Polysaccharide-Based Magnetic Gel for Direct Red 83:1 Removal from Water
Source: Gels. 2024 Jul 26;10(8):496. doi: 10.3390/gels10080496 (PMC11354028; doi:10.3390/gels10080496)
Supplement: Supplementary file 1 [file gels-10-00496-s001.zip › gels-3088981-supplementary.pdf]

Supplementary Material for "Development of chitosan polysaccharide-based magnetic gel for Direct Red 83:1 removal from water"

Ainoa Murcia-Salvador [1], María Isabel Rodríguez-López [1], José Antonio Pellicer [1], Teresa Gómez-Morte [1], David Auñón [1], María Josefa Yáñez-Gascón [1], José Pedro Cerón-Carrasco [2], Ángel Gil-Izquierdo [3], Estrella Núñez-Delicado [1] and José Antonio Gabaldón [1],*

Affiliations:

[1]Molecular Recognition and Encapsulation Research Group (REM), Health Sciences Department, Univer-sidad Católica de Murcia (UCAM), Campus de los Jerónimos 135, Guadalupe, E-30107, Spain.
[2]Centro Universitario de la Defensa, Universidad Politécnica de Cartagena, C/Coronel López Peña s/n, Base Aérea de San Javier, Santiago de la Ribera, E-30720, Spain.
[3]Research Group on Quality, Safety and Bioactivity of Plant Foods, Department of Food Science and Technology, CEBAS-CSIC, University Campus of Espinardo-Edif. 25, 30100 Espinardo, Spain.

*Corresponding author: José Antonio Gabaldón (jagabaldon@ucam.edu)

Supplemental figures

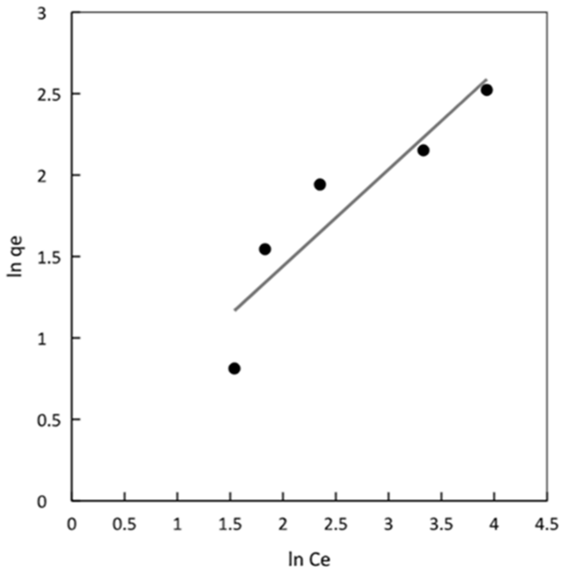

Figure S1. Freundlich isotherm plots for chitosan magnetic adsorbents.

.

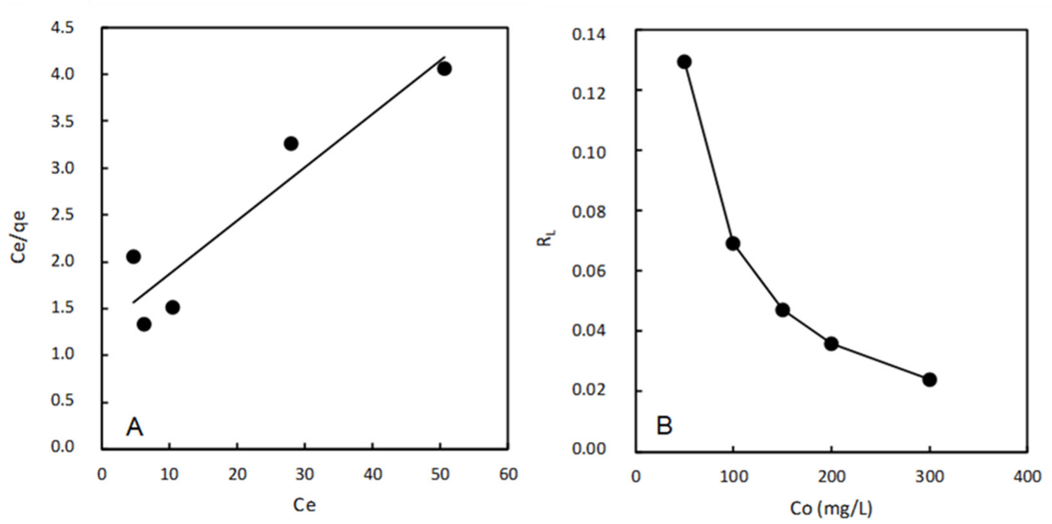

(a) (b)

Figure S2. (a) Langmuir isotherm plots. (b) Separation factor.

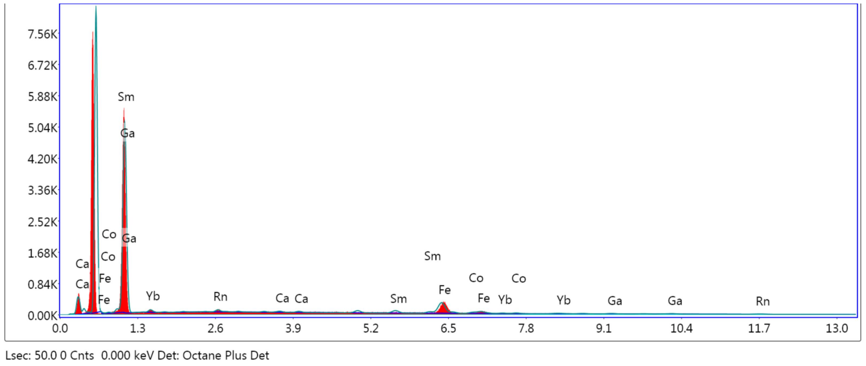

(a)

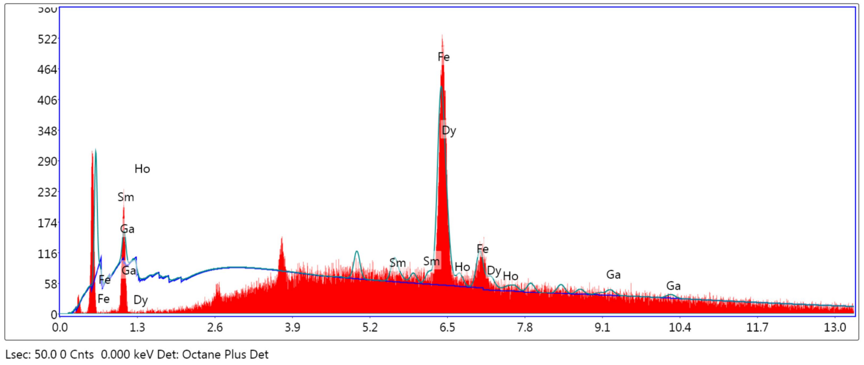

(b)

Figure S3. (a) EDX of Chitosan and (b) Chitosan-Fe magnetic polymer.

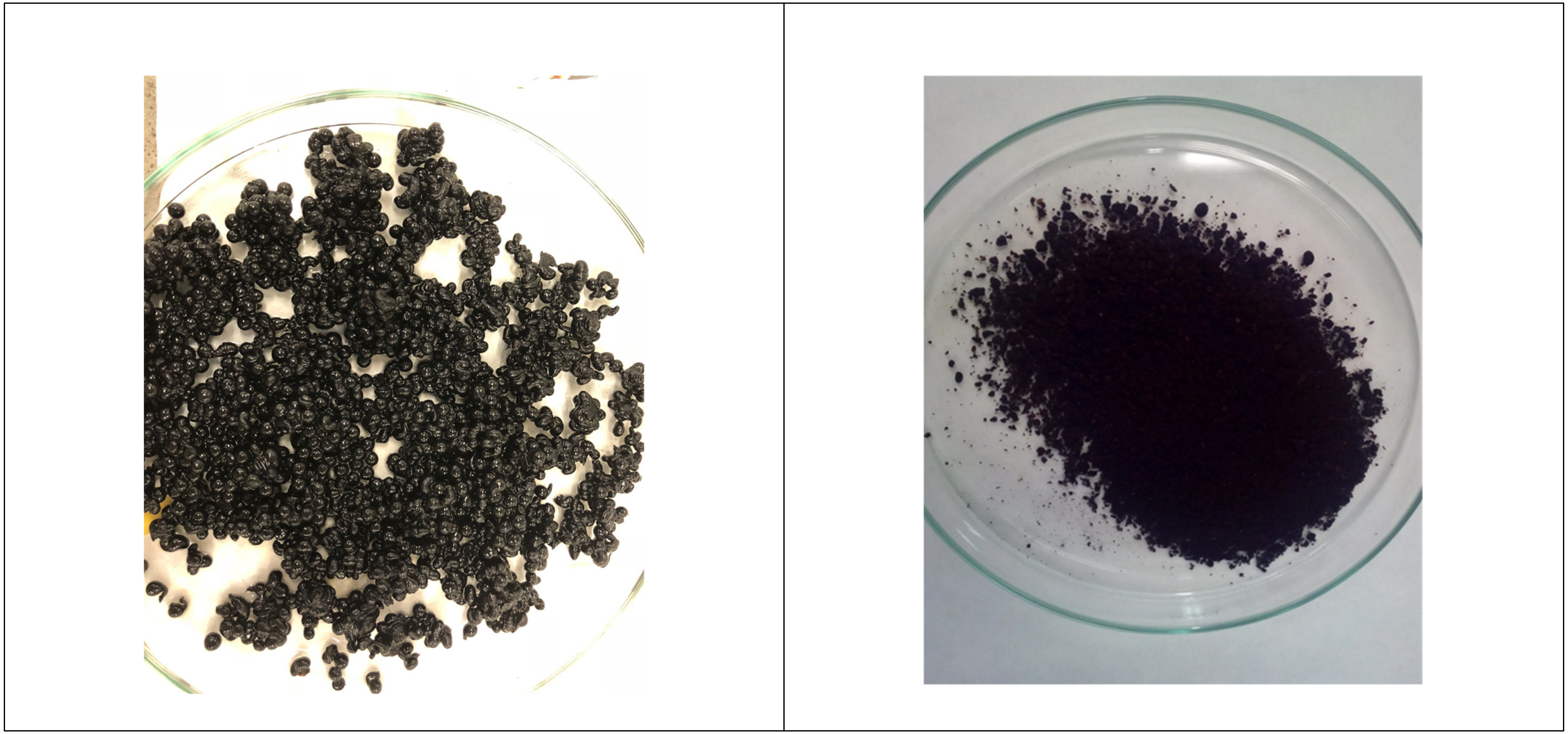

(a) (b)

Figure S4. Chitosan magnetic polymeric gel beads. (a) before drying process, (b) after drying process

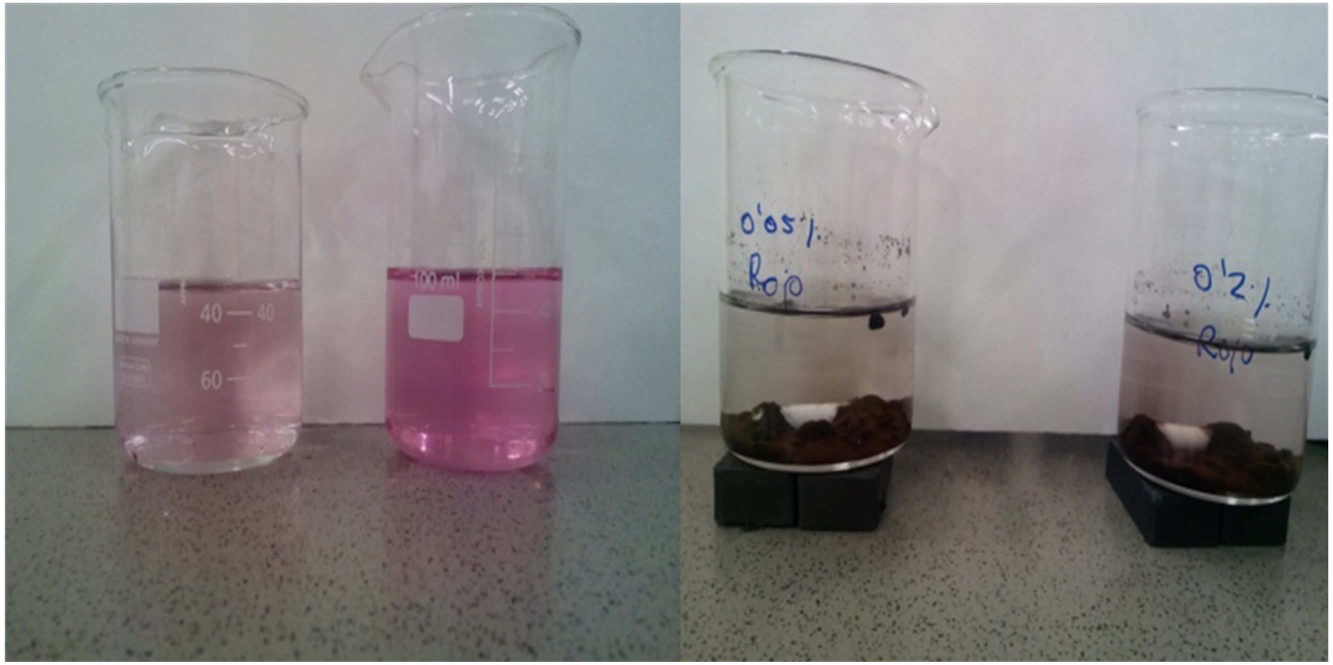

(a) (b)

Figure S5. Adsorption tests. (a) Solutions containing 50 mg/L and 200 mg/L of Direct Red 83:1. (b) Separation of the magnetic polymer (1g) after 10 minutes of contact time, by influence of an external ferrite magnet for 5 minutes.

Supplemental table

Table S1. Kinetic parameters (pseudo 1st, pseudo 2nd and intraparticle diffusion models) for adsorption of Direct Red 83:1 onto chitosan magnetic polymeric gel.

**PFOM[1]**		**Chitosan-Fe**		
C_0 (mg/L)	qe_{exp}	qe_{cal}	$K_1 (min^{-1})$	R^2
50	2.27	1.05	0.033	0.987
100	4.69	3.11	0.040	0.881
150	6.97	5.31	0.031	0.960
200	8.60	9.36	0.027	0.950
300	12.47	18.29	0.029	0.772
**PSOM[2]**		**Chitosan-Fe**		
C_0 (mg/L)	qe_{exp}	qe_{cal}	$K_2 (min^{-1})$	R^2
50	2.27	2.27	0.075	0.999
100	4.69	4.69	0.033	0.999
150	6.97	6.97	0.014	0.999
200	8.60	8.60	0.009	0.999
300	12.47	12.47	0.007	0.999
**IDM[3]**		**Chitosan-Fe**		
C_0 (mg/L)	qe_{exp}	qe_{cal}	K_i (mg/g $min^{1/2}$)	R^2
50	2.27	1.06	0.11	0.930
100	4.69	1.50	0.32	0.662
150	6.97	2.08	0.46	0.834
200	8.60	0.63	0.71	0.978
300	12.47	-1.37	1.20	0.977

[1]PFOM: Pseudo-first-order model; [2]PSOM: Pseudo-second-order model; [3]IDM: Intraparticle diffusion model.
